# Anti-Inflammatory Effect of Muscle-Derived Interleukin-6 and Its Involvement in Lipid Metabolism

**DOI:** 10.3390/ijms22189889

**Published:** 2021-09-13

**Authors:** Hidetoshi Nara, Rin Watanabe

**Affiliations:** Department of Biological Sciences, Faculty of Science and Engineering, Ishinomaki Senshu University, Ishinomaki 986-8580, Japan; ml210104@edu.isenshu-u.ac.jp

**Keywords:** IL-6, myokine, exercise, soluble IL-6R, gp130, NAFLD

## Abstract

Interleukin (IL)-6 has been studied since its discovery for its role in health and diseases. It is one of the most important pro-inflammatory cytokines. IL-6 was reported as an exacerbating factor in coronavirus disease. In recent years, it has become clear that the function of muscle-derived IL-6 is different from what has been reported so far. Exercise is accompanied by skeletal muscle contraction, during which, several bioactive substances, collectively named myokines, are secreted from the muscles. Many reports have shown that IL-6 is the most abundant myokine. Interestingly, it was indicated that IL-6 plays opposing roles as a myokine and as a pro-inflammatory cytokine. In this review, we discuss why IL-6 has different functions, the signaling mode of hyper-IL-6 via soluble IL-6 receptor (sIL-6R), and the involvement of soluble glycoprotein 130 in the suppressive effect of hyper-IL-6. Furthermore, the involvement of a disintegrin and metalloprotease family molecules in the secretion of sIL-6R is described. One of the functions of muscle-derived IL-6 is lipid metabolism in the liver. However, the differences between the functions of IL-6 as a pro-inflammatory cytokine and the functions of muscle-derived IL-6 are unclear. Although the involvement of myokines in lipid metabolism in adipocytes was previously discussed, little is known about the direct relationship between nonalcoholic fatty liver disease and muscle-derived IL-6. This review is the first to discuss the relationship between the function of IL-6 in diseases and the function of muscle-derived IL-6, focusing on IL-6 signaling and lipid metabolism in the liver.

## 1. Introduction

To date, many cytokines have been discovered and they work intricately through crosstalk in the body. Cytokines exert their effects on cells via autocrine, paracrine, and endocrine signaling, which result in cell proliferation, differentiation, and death, among other outcomes. In addition, cytokines can control the immune system [1]. Interleukins (ILs) are a group of cytokines, with over 40 types reported so far [2]. Among them, IL-6 is classified as a pro-inflammatory cytokine [3]. IL-6 is secreted by T cells and was identified as an essential cytokine for B cell terminal differentiation [4]. At present, it is known that the production of somatic cells, such as fibroblasts [5], epidermal cells [6], and muscles [7], involves macrophage activity [8,9]. IL-6 belongs to the cytokine superfamily that uses glycoprotein 130 (gp130) as a signal-transducing receptor. Its family includes IL-11, IL-27, IL-31, oncostatin M, leukemia inhibitory factor, ciliary neurotrophic factor, cardiotrophin 1, and cardiotropin-like cytokine factor [10,11]. It was reported that infections induced with *Listeria* species are exacerbated in IL-6 knockout mice without causing an acute inflammatory reaction [12]. Furthermore, the rapid production of IL-6 plays an important role in defense mechanisms against infections, while excessive production of IL-6 results in various defects. Rheumatoid arthritis is a disease that is associated with excessive IL-6 levels; therefore, IL-6 signaling molecules were targeted in the treatment of rheumatoid arthritis [11,13,14,15]. Consequently, IL-6 has attracted attention for its function as a pro-inflammatory cytokine; however, in recent years, it has become difficult to understand the functions of IL-6.

Exercise helps to prevent the development of various illnesses [16,17]. For instance, it is effective in the prevention of type 2 diabetes mellitus (T2DM) [16,18], as well as its therapy [19]. Additionally, it is reported that exercise helps to prevent breast cancer [20], cardiovascular mortality [21,22], colon cancer [23], and sarcopenia [24] by improving lipid metabolism, glucose metabolism, and insulin resistance [25,26,27]. Exercise involves the contraction of skeletal muscle, and since the contraction results in the secretion of physiologically active substances, skeletal muscle is an endocrine organ. Consequently, the substances secreted by the skeletal muscle are termed myokines [7,28,29]. There are reports on several hundreds of myokines [30]. IL-6 is one of the most important myokines. It was indicated that the function of muscle-derived IL-6 is not linked to pro-inflammation but to lipid metabolism. Since the skeletal muscle is the largest organ in the human body and accounts for 40% of total body weight, its effects on health as an endocrine organ cannot be ignored. Understanding the conflicting functions of IL-6 will be necessary not only for the correct therapy for COVID-19 but also for us to live healthy and enjoyable lives.

## 2. IL-6 as a Myokine

It is well known that moderate exercise is good for the body. However, the reason has not been explained based on clear scientific evidence. This is because exercise involves not only skeletal muscle contraction but also systemic activities of the respiratory and circulatory systems. Furthermore, since various cells, such as fibroblasts and vascular endothelial cells, are localized in skeletal muscle tissue, it is difficult to perform molecular analyses to assess the effects of exercise. However, the mouse myoblast cell line C2C12 is an effective model culture system that can be used for such analyses to solve this problem. The C2C12 cell line has been classically used to study myoblast proliferation and differentiation since the mid-1980s and is still an important tool today [31,32]. In recent years, studies on systems that artificially contract C2C12 cells using electrical stimulation were actively used, and many findings were reported [33,34,35,36]. It was reported that electrical stimuli can cause C2C12 cells that have been induced to form myotubes to secrete cytokines (previously identified as myokines), such as IL-6 and IL-15 into the culture supernatant [33].

We have also previously reported that myoblast-derived cytokines act in an autocrine/paracrine manner in primary bovine myoblasts [37,38]. These studies were performed based on the hypothesis that cytokines that are produced by the skeletal muscles are involved in activities that affect muscle physiology, such as regeneration and hypertrophy. In autocrine/paracrine interactions, the effects on systemic tissues are weak and it is necessary for myokines to be secreted into blood vessels.

Increased levels of calcium ions in the muscles due to contraction result in the activation of p38 mitogen-activated protein kinase (MAPK) and calcineurin to secrete IL-6 [39]. It was reported that exercise significantly increases IL-6 secretion in humans. In a previous study, the changes in cytokine levels in blood after a marathon were found to be remarkable [40]. In the study, the levels of some inflammatory cytokines (tumor necrosis factor alpha (TNFα), IL-1β, and IL-6) and anti-inflammatory factors (IL-1ra, IL-10, and sTNF receptors 1 and 2) were measured every thirty minutes after a marathon in men aged 24–37 years. Among the measured parameters, IL-6 and IL-10 showed markedly increased levels in blood. It was indicated in another report that IL-6 and IL-10 levels in blood are increased immediately after a marathon [41]. Furthermore, the mRNA expression of IL-6 and IL-10 is reportedly increased when C2C12 cells are stimulated with calcium ionophores, such as A23187 and IBS008738, which regulate the expression of the transcription factor myoblast determination protein 1 during myoblast differentiation [42]. Interestingly, the pro-inflammatory cytokine IL-6 and the anti-inflammatory cytokine IL-10 show similar tendencies in such cases.

It was reported that calcium ions are released from the sarcoplasmic reticulum into the cytoplasm during a muscle contraction and that the contraction itself, not the calcium ions, is important for IL-6 secretion [34]. An A23187-induced increase in calcium ion concentration in the cytoplasm and a muscle contraction are different phenomena [43]. Therefore, it is difficult to interpret an A23187-induced increase in the mRNA expression of IL-6. Our research group also confirmed that the mRNA expression of IL-6 is 100-fold higher in myotubes than in proliferating myoblasts and that it is enhanced (about 1000-fold) after A23187 stimulation (R. Watanabe, unpublished data).

A high glucose status is known to be an exacerbating factor for pancreatic [44], breast [45], and bladder [46] cancers. Muscle-derived IL-6 was found to enhance insulin sensitivity in the plantaris muscle of mice by enhancing the expression of glucose transporter 4 (GLUT4). However, this effect was canceled after the mice were injected with an IL-6-neutralizing antibody before exercise [47]. The expression of GLUT4 peaked in both the plantaris and soleus muscles when IL-6 concentration was increased from 100 to 1000 pg/mL, which was due to the direct injection of IL-6 into the blood of the mice. This suggests that IL-6 as a myokine has a tumor-suppressing effect. Muscle exercise increases plasma IL-6 levels up to 100-fold and causes high glucose consumption. Moreover, during exercise, it is considered that IL-6 causes tissues to take up glucose before it is depleted. Muscle damage that is caused by strenuous exercise results in an increased IL-6 level in the blood. Initially, it was thought that this IL-6 was derived from monocytes, mainly macrophages, for tissue repair; however, it was revealed to be derived from the muscles [48]. IL-6 production from macrophages and monocytes is mediated by toll-like receptors and the nuclear factor kappa B pathway [49,50]. In skeletal muscles, contraction and glycogenosis result in increased p38 MAPK activation, which then promotes IL-6 mRNA transcription [7]. These phenomena have caused some confusion, as IL-6 has been considered an inflammatory cytokine. Recently, IL-6 has been considered as a factor that exacerbates the symptoms of coronavirus disease (COVID-19) [51,52,53,54]. Therefore, it is very important to clarify whether monocyte-derived IL-6 has a pro-inflammatory effect and muscle-derived IL-6 has an anti-inflammatory effect, or whether some other factors are at play in these contrasting effects of IL-6.

## 3. Classical Signaling and *Trans*-Signaling of IL-6

IL-6 binds to the specific receptor IL-6R (CD126) to form an IL-6/IL-6R complex, which causes homodimerization of the signal component gp130 (CD130). The intracellular domain of IL-6R has only 82 amino acids and does not have a signal transduction mechanism by itself. In contrast, gp130 has 277 amino acids and a cytoplasmic domain containing phosphorylation sites. IL-6 activates molecules that are involved in intracellular signal transduction, such as Janus kinase 1 (JAK1), JAK2, and tyrosine kinase 2, as well as downstream signaling molecules, such as signal transducer and activator of transcription 3 (STAT3), STAT1 [55,56,57], and phosphatidylinositol 3-kinase. It also activates molecules that are involved in MAPK downstream signaling pathways [58]. STAT3 causes negative feedback and promotes the transcription of suppressor of cytokine signaling 1 (SOCS1) and SOCS3. In addition, SOCS1 inhibits JAK activity, SOCS3 binds to gp130 and inhibits Src homology domains containing tyrosine phosphatase-2 pathway upstream of the MAPK pathway [59,60,61,62]. The classical IL-6 signaling system is a *cis*-signaling (classical signaling) system in which all receptor components are present on the same cell surface, and many cytokines take that form. In addition, IL-6 was reported to take the form of *trans*-signaling [63,64,65]. It is reported that sIL-6R binds to IL-6 to form a complex named hyper-IL-6 [66], which also binds to gp130. There are similar interesting reports that classical IL-6 signaling and *trans*-signaling work quite differently. It was shown that inflammation of retinal endothelial cells and associated barrier disruption are suppressed via the inhibition of IL-6 *trans*-signaling [67]. Furthermore, in the management of COVID-19, tocilizumab, which prevents sIL-6 and membrane-bound IL-6R from forming a complex with gp130, inhibits the pro- and anti-inflammatory pathways. Consequently, IL-6 inhibitors should be cautiously used to manage COVID-19 [51,68,69]. The classical IL-6 signaling system requires membrane-bound IL-6R, whose expression is limited to macrophages, neutrophils, T cells, and hepatocytes [70]. It was reported that two mechanisms lead to the generation of sIL-6R. One mechanism is the alternative splicing of IL-6R variants that lack the transmembrane domain [71,72]. The other is the proteolytic cleavage of mbIL-6R, which is dependent on the activity of a disintegrin and metalloprotease (ADAM). The ADAM family metalloproteases are involved in the proteolysis of adhesion molecules, cytokines/chemokines, growth factors [73], and many cytokine receptors, such as TNFR, IL-1R, and IL-6R [74,75]. IL-6R is a substrate of ADAM10 and ADAM17 [76], which both belong to the transmembrane ADAMs family [77]. ADAM10 is responsible for the slow constitutive release of IL-6R, whereas ADAM17 causes rapid proteolysis of IL-6R [78]. Despite the limited expression of IL-6R, gp130 is expressed in all cells; therefore, IL-6 *trans*-signaling has systemic effects. Phorbol 12-myristate 13-acetate was used as an ADAM17 activator in a previous in vitro study [71]. It is also reported that in vivo cholesterol depletion induces IL-6R shedding from murine fibroblasts and human monocytes by ADAM17 [78,79].

It is necessary to consider whether sIL-6R is involved in IL-6 activity during muscle contraction. In chronic heart failure patients, 12 weeks of programmed exercise was found to reduce the sIL-6R level in plasma [80]. Furthermore, the plasma levels of sIL-6R tended to rise for six months in obese postmenopausal women on a low-calorie diet alone. However, plasma sIL-6R levels significantly decreased over three days of walking on a treadmill [81]. Gray and collaborators also evaluated the serum level of IL-6/sIL-6R in healthy young males after one hour of cycling exercise. The authors revealed that the blood levels of both IL-6 and sIL-6R increased after the exercise. Furthermore, it was shown that the level of the IL-6/sIL-6 complex increased by 2.1 times immediately after exercise and 1.8 times after 1.5 h of exercise [82]. These facts indicate that exercise has some effects on the secretion of sIL-6R, and that the effects are different depending on the type of exercise. It was shown that cycling is a strenuous exercise that results in increased sIL-6R secretion [83]. Generally, mild exercise is better for health than strenuous exercise is, which may be due to the effects of sIL-6R secretion into plasma. Future studies are needed to clarify the mechanism of ADAM10 and ADAM17 activation that leads to sIL-6R secretion in skeletal muscles.

**Figure 1:**
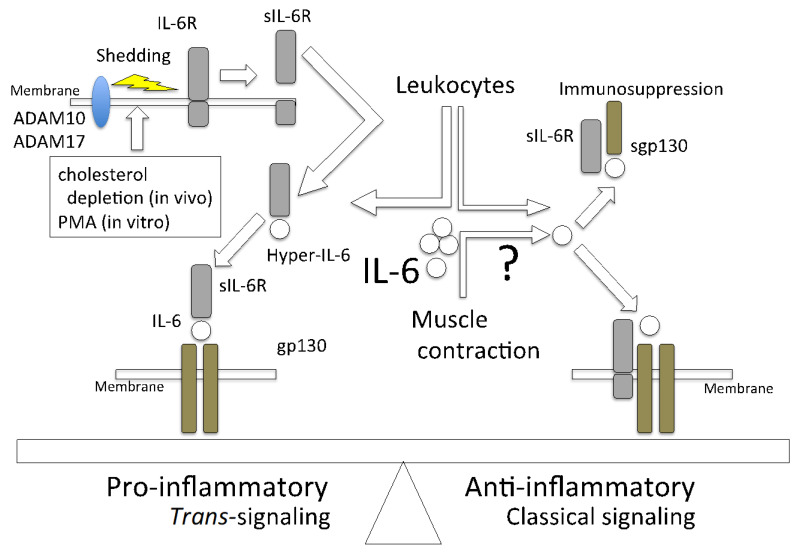
IL-6 shows a pro- or anti-inflammatory effect that is determined by its signaling manner. The pro-inflammatory function of IL-6 is due to *trans*-signaling. This occurs when IL-6R (sIL-6R) is cleaved by sheddases, such as ADAM10 and ADAM17, which are activated by the depletion of cholesterol and/or phorbol 12-myristate 13-acetate (PMA) into its secretory form and binds to IL-6, which is followed by signaling to gp130-expressing cells. The anti-inflammatory effect is induced in the classical signaling manner. Although sgp130 has a low affinity for IL-6 alone, it has a high affinity for the IL-6/sIL-6 complex (hyper-IL-6); therefore, it suppresses the pro-inflammatory effect.

IL-6-induced pro-inflammatory response is inhibited by soluble gp130 (sgp130), which is generated from alternative splicing [64]. Specifically, sgp130 inhibits IL-6 *trans*-signaling but not classical IL-6 signaling [84]. Although gp130 has a low affinity for IL-6, it shows a high affinity for IL-6 in the presence of IL-6R. This is reportedly the case because sgp130 inhibits pro-inflammation by trapping hyper-IL-6; however, it does not act on sIL-6 alone, which causes classical signaling to be activated. It is reported that exercise suppresses weight gain to some extent in wild-type mice that are fed a high-fat diet (HFD), whereas weight gain is not suppressed in adipocyte-specific gp130-deficient mice, even if they exercise [85]. Furthermore, in endothelial cells and CD3^+^ T cells, the activation of STAT3 by hyper-IL-6 is suppressed in a concentration-dependent manner by sgp130 [86]. Additionally, artificially synthesized sgp130 (sgp130-fc) was shown to be useful in many mouse models of human diseases, such as cancer, rheumatoid arthritis, sepsis, and asthma [87]. The molecules involved in IL-6 signaling are shown in Figure 1.

## 4. Hyper-IL-6 and sgp130 Production during Exercise

The contradicting observations showing that the IL-6 secreted during exercise has an anti-inflammatory effect whereas leukocyte-derived IL-6 has a pro-inflammatory effect may be related to the plasma levels of sIL-6 and sgp130. The plasma concentrations of IL-6, sIL-6R, and sgp130 in patients with juvenile idiopathic arthritis were measured after twenty minutes of exercise in a previous study [88]. The results showed increases in IL-6 and sgp130 levels after the exercise. In contrast, plasma sIL-6R concentration decreased by about 8.4% after exercise [88]. Another group reported increased IL-6 production but decreased plasma levels of sIL-6R and sgp130 after both high-intensity and moderate-intensity exercise in overweight males (29.0 ± 3.1 kg/m^2^) [89]. The expression level of gp130 in skeletal muscle changes with exercise. In another study, exercise training resulted in a significant increase in the protein expression of muscle gp130 in B6 mice, whereas STAT3 phosphorylation was significantly reduced in the gastrocnemius muscle of the mice [90]. It is interesting that STAT3 activation reduced in the exercise group but gp130 expression increased. It is difficult to interpret this seemingly contradictory result, as it is presumed that the blood level of sIL-6R is reduced after exercise.

Hyper-IL-6 production requires the secretion of sIL-6R by ADAM10 and/or ADAM17. There are few reports on the relationship between exercise and the activation of ADAM family proteins and/or their expression on IL-6R-expressed cells. In one study, it was found that exercise reduces the activation of ADAM10 and/or ADAM17, and that the expression levels of these two ADAMs did not change after exercise in peripheral blood mononuclear cells [91]. At present, there is no report that directly indicates that exercise suppresses the activation of ADAM10 or ADAM17 and IL-6R shedding. Although sgp130 is effective in suppressing hyper-IL-6 signals, it is difficult to study the synthetic pathway of sgp130.

There are few reports on ADAMs in muscle tissue; however, two studies clearly showed that ADAM10 is expressed on muscle cells [92,93]. In order to repair muscle damage caused by excessive exercise or trauma, the proliferation of muscle-specific satellite cells in the muscle fibers and then differentiation are required. ADAM10 in muscle satellite cells maintains the satellite cell pool; therefore, the inhibition of ADAM10 activity accelerates satellite cell differentiation [93]. The expression of ADAM17 in muscle tissue was confirmed and seems to be related to hypoxia [94].

There are three sgp130 isoforms [95]: sgp130-RAPS [96], sgp130-E10 [97], and full-length sgp130 [98]. Unfortunately, there is no information on the secretion of these isoforms during exercise. Additionally, the genes that regulate sgp130 levels are largely unknown [95].

## 5. Effects of IL-6 Concentration on Its Activity

The IL-6 concentration in blood is approximately 4 pg/mL; however, this value increases to microgram per milliliter levels during septic shock and cytokine storms [14]. The response to IL-6 in the C2C12 myoblast cell line is interesting. It is reported that the extent of activation of molecules involved in intracellular signal transduction by recombinant IL-6 is dose-dependent, differing between a low dose of 10 pg/mL and a high dose of 10 ng/mL. IL-6 phosphorylates JAK1 and promotes cell proliferation at a low dose, whereas it promotes JAK2 activation and accelerates differentiation of myoblasts into myotubes at a high dose. In another study, IL-6 had different effects on the expression of SOCS family molecules. Specifically, low-dose and high-dose IL-6 induced the expression of SOCS1 and SOCS3, respectively [99]. SOCS1 directly binds to JAKs, whereas SOCS3 binds to phosphorylated tyrosine residues of activated cytokine receptors to inhibit signaling [100]. Additionally, SOCS1 suppresses all the activities of downstream molecules of cytokine receptors, while SOCS3 preferentially inhibits the JAK-STAT pathway. The reasons for this difference in the inhibitory effects of these molecules, and whether proliferation and differentiation are involved, must be investigated in future studies. Currently, there are no reports on the dose-dependent effect of IL-6 on signal transduction in other cells. C2C12 cells also express IL-6R and gp130; however, little is known about the expression of ADAM family proteins on the cells. Only one report has indicated that ADAM12 is expressed on C2C12 cells [101]. Therefore, it is considered that the difference in signaling is not due to sIL-6R but purely due to IL-6 concentration. It is possible that these effects may be different in future in vivo studies depending on the balance between the concentrations of IL-6 and sIL-6R.

## 6. Role of IL-6 in Liver Disease and Lipid Metabolism

### 6.1. Role of IL-6 in Liver Disease

There are many findings on the effects of IL-6 on the liver. The activation of STAT3 by IL-6 results in macrophage polarization into M1 macrophages, which are involved in the development of hepatocellular carcinoma (HCC) [102]. The blockade of IL-6 and programmed death-ligand 1 (PD-L1) activities resulted in the inhibition of HCC development. Additionally, IL-6 produced by cancer-associated fibroblasts induces immunosuppression by recruiting myeloid-derived suppressive cells and enhancing PD-L1 expression [103]. Pro-inflammatory cytokines, such as TNFα, IL-1β, and IL-6, contribute to the activation and recruitment of Kupffer cells, which are resident macrophages in the liver [104]. Increased IL-6 levels and overactivated STAT3 were observed in HCC patients [105,106]. The role IL-6 from Kupffer cells is unclear. It was demonstrated that serum IL-6 level is higher in HCC patients. Particularly, it is lower in grades II and III patients (large number of tumors) than in grade I (small number of tumors) patients. It was also shown in the study that tumor size in the liver was smaller in monocyte-specific IL-6-deficient mice than in wild-type mice. The IL-6/JAK/STAT3 pathway is crucial for carcinogenesis, as it increases the protein levels of B-cell lymphoma-extra large, cyclin D1, and c-Myc [107]. IL-6 upregulates androgen receptors, which results in the reduced expression of the tumor suppression molecule p53 and increased production of reactive oxygen species [108].

There are also reports on PM2.5, which has become a problem in recent years, and liver diseases. It has been shown that exposure to PM2.5 results in a significant increase in the blood IL-6 level but not the TNFα level. IL-6 then activates the STAT3/SOCS3 pathway in the liver, resulting in decreases in the expression of GLUT2 and GLUT4 in hepatocytes, which is the cause of type 2 diabetes [109].

Conversely, IL-6 has a beneficial role in liver regeneration. In a study that was conducted on mice, IL-6 *trans*-signaling promoted liver regeneration after partial hepatectomy (PHX) was performed [110]. Hepatocyte growth factor (HGF), which is secreted by liver satellite cells, is the most important cytokine for the proliferation of liver cells [111]. In PHX, sIL-6R plays an important role in HGF production. Our previous in vitro data showed that the culture supernatant of C2C12 myoblasts, which express both IL-6R and gp130, enhanced the proliferation of Hepa1–6 cells, whereas recombinant IL-6 itself showed no effect (R. Watanabe, unpublished data). Therefore, we believe that by clarifying the trends of sgp130, HGF, sIL-6R, ADAM10, and ADAM17 expression, such an in vitro experimental system will be an effective model for studying liver injury.

### 6.2. Role of Muscle-Derived IL-6 in Lipid Metabolism in Adipocytes

It was reported that gp130-mediated signals in adipocytes are involved in weight loss [85]. Furthermore, exercise does not induce the activation of STAT3, JAK, or Akt in adipocyte-specific gp130-deficient mice that are fed an HFD. Moreover, like adipocytes, hepatocytes have a high ability to accumulate lipids. In muscle-specific IL-6-deficient mice, the expression of phosphoenolpyruvate carboxykinase, which is related to glyceroneogenesis, was 1.3-fold higher than that in wild-type mice [112]. IL-6 derived from skeletal muscle has some effect on lipolysis and glyceroneogenesis in adipose and hepatic tissues [112]. It was also shown in several studies that lipid metabolism occurs in muscle-specific IL-6-deficient mice (IL-6 MKO) [113,114,115]. In a previous study in IL-6 MKO and wild-type mice on an HFD, it was found that inguinal and epididymal white adipose tissue weights reduced relative to body weight; however, caloric intake was higher in the IL-6 MKO mice than in the wild-type mice. Interestingly, this difference between the two groups of mice disappeared after the animals exercised. Additionally, GLUT4 expression, phosphorylation of 5′ AMP-activated protein kinase (AMPK), and the mRNA level of fatty acid synthetase were lower in the IL-6 MKO mice [113]. AMPK inhibits the activation of sterol regulatory element-binding protein 1, which is a master transcriptional regulator of lipid synthesis [116]. The relationship between exercise and AMPK activation was also revealed in an experiment performed in mice. In the study, AMPK levels after exercise were lower in adiponectin-deficient mice for 15 days than in wild-type mice [117]. In another experiment, increases in the concentrations of IL-6 and IL-10 in blood after exercise were also more suppressed in adiponectin-deficient mice than in wild-type mice. Adiponectin is an adipokine with anti-inflammatory, antioxidant, and insulin-sensitizing properties [118]. Thus, the data show that muscle-derived IL-6 directly affects lipid metabolism in adipose tissue.

## 7. Role of IL-6 Signaling in Nonalcoholic Fatty Liver Disease (NAFLD)

### 7.1. Total IL-6 on NAFLD

NAFLD is a general term for a fatty liver that is caused by factors other than alcohol. It is the most common form of chronic liver disease worldwide [119]. NAFLD is mostly accompanied by obesity, diabetes, dyslipidemia, and hypertension; therefore, it is considered a liver disease of metabolic syndrome. It was reported that NAFLD can progress to nonalcoholic steatohepatitis (NASH), cirrhosis, and HCC [119]. The two-hit theory for NAFLD onset was described by Adams et al. [120]. Lipids accumulate in the liver due to disordered or biased eating habits, such as the intake of high-fat and high-energy diets, which is the first hit. In the second hit, the state of the liver results in the development of NASH, which is due to the effects of oxidative stress and inflammatory cytokines. At this stage, there is an increase in the levels of several cytokines, particularly IL-6. However, the role of IL-6 in the onset of NAFLD is unclear [121]. Lipid droplets are accumulated in the livers of systemic-IL-6-deficient mice. This is because the classical IL-6 signaling pathway in macrophages results in the secretion of exosomes containing microRNA-223 (miR-233), which suppresses the activity of NLRP3 inflammasome in hepatocytes. As a result, inflammation is suppressed and NAFLD-associated fibrosis is attenuated [121]. These findings provide the therapeutic target for NAFLD. It was also found that serum IL-6 levels in NAFLD patients and serum sIL-6R levels in NASH patients are significantly higher than their respective normal levels. Other researchers also showed that miR-233 ameliorates oxidative stress and insulin resistance in HepG2 cells treated with a high glucose concentration [122]. However, the role of miR-233 in liver disease remains unclear. It was reported that miR-233 deficiency protects against Fas-induced hepatocyte apoptosis and liver injury by targeting insulin-like growth factor 1 [123].

Furthermore, Skuratovskaia et al. found that the plasma IL-6 level was threefold higher, whereas plasma sgp130 level was one-third lower in obese patients with T2DM than their respective levels were in healthy and obese patients without T2DM [124]. Additionally, although the sIL-6R levels were unexpectedly lower in obese patients with T2DM than in the other groups of patients, there is the possibility that hyper-IL-6 was involved in the observed effects in obese patients with T2DM. Moreover, the gene expression of superoxide dismutase (SOD) type 1 in the liver was lower in T2DM patients than it was in healthy control patients. In contrast, the number of infiltrating lymphocytes and the area of steatosis were higher in the T2DM patients. It was also reported that SOD reduces damage to cells by suppressing the oxidation of lipids that are associated with NAFLD [117].

### 7.2. Muscle-Derived IL-6 Directly Decreases Lipid Droplets via Autophagy

**Figure 2:**
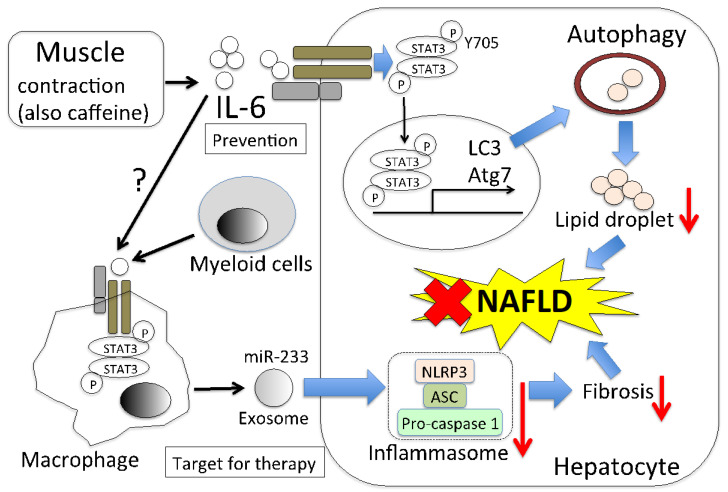
Predicted NAFLD alleviation model of muscle-derived IL-6. Muscle contraction promotes IL-6 production from muscle cells. The produced IL-6 is then secreted into the blood and it induces the phosphorylation of STAT3 in hepatocytes. Phosphorylated STAT3 translocates into the nucleus. Autophagosomes are formed due to the activity of autophagy-related genes, such as LC3 and Atg7, whose expression is regulated by phosphorylated STAT3 (Y705). Autophagy results in the breakdown of lipids in hepatocytes, leading to NAFLD alleviation. There is also a pathway in which IL-6 that is derived from myeloid cells reduces fibrosis in hepatocytes. This is the pathway by which IL-6 acts on macrophages to secrete exosomes, including miR-233. The miR-233 reduces the inflammasome activity of hepatocytes. Whether muscle-derived IL-6 has a similar effect is unclear. Thus, the muscle-derived IL-6 signaling pathway is important in the prevention of NAFLD, and leukocyte-derived IL-6 is important from a therapeutic point of view.

It is very important to know whether muscle-derived IL-6 affects hepatic lipids from the perspective of preventing NAFLD. Caffeine is found in coffee and tea and is taken by many people through various foods. There are research reports that caffeine may have a protective effect against NAFLD and reduce the levels of aspartate aminotransferase and alanine aminotransferase [125,126,127,128]. The underlying mechanism was investigated and it was found that autophagy results in reduced lipid levels in the liver [129,130,131]. Although animal experiments and cohort studies showed that caffeine is effective at suppressing NAFLD development, the effects of caffeine on hepatocytes are still controversial [126,131]. It was also reported that caffeine does not act directly on hepatocytes and macrophage, but on muscles. Caffeine-stimulated myocytes produce IL-6, which activates STAT3 phosphorylation (Tyr705) in hepatocytes. This results in the expression of microtubule-associated protein light chain 3 (LC3) and autophagy-related 7 (Atg7), which are autophagy-related molecules [130]. Hepatic autophagy maintains metabolic homeostasis in lean mice, but Atg7 expression was reported to be impaired in HFD-fed mice [131,132]. In mice that are HFD-fed and caffeine stimulated, LC3 and Atg7 expressions are maintained, whereas, in IL-6- or STAT3-deficient mice, the effect of caffeine for the expression of LC3 and Atg7 was not observed [130]. Interestingly, this IL-6 production occurs without muscle contraction, but caffeine intake together with exercise increases the amount of IL-6 in plasma more than exercise alone does [133]. Furthermore, caffeine-treated mice on HFD showed lower levels of hepatic triglycerides, both histologically and biochemically, than mice that were only on an HFD did in a previous study [131]. Thus, it is clear that muscle-derived IL-6 affects NAFLD improvement, indicating that it could be a direct target in the treatment of NAFLD. The predicted model of NAFLD alleviation by muscle-derived IL-6 is shown in Figure 2.

Yu et al. reported pyruvate kinase M2 inhibits autophagy via JAK/STAT3 pathway in HC [134]. There is a contradiction in how STAT3 works with respect to autophagy. The contradiction may be due to the difference between normal cells and cancer cells or the difference in the phosphorylated region of STAT3. There are two phosphorylated regions of STAT3, Ser727, and Tyr705. Fang et al. evaluated only the phosphorylation of Tyr705 in hepatocytes. Whether muscle-derived IL-6 is involved in the phosphorylation of Ser727 is an interesting issue.

## 8. Conclusions and Prospects

Muscle-derived IL-6 does not act as an inflammatory cytokine, but rather as an anti-inflammatory cytokine. It is speculated that the different functions of IL-6 are due to the signaling system of IL-6, which involves the secretion of sIL-6 and sgp130. It is necessary to analyze the mechanism by which these molecules are secreted, including how ADAM10 and ADAM17 sheddases are activated. In addition, downstream IL-6 signaling molecules that are transcribed differ depending on IL-6 concentration. Thus, the complex molecular mechanisms underlying sIL-6R and sgp130 production and IL-6 concentration may be involved in IL-6 signaling, which makes the discussion more complicated. There are many reports on the function of IL-6 as a myokine; however, this review was focused on lipid metabolism, especially in the liver. Other events and processes may be used to clarify the current data; therefore, further studies on this subject are needed. Since the development of molecular-targeted drugs that have the same effect as exercise is expected for humans who have difficulty exercising, it is expected that the mechanism of action of muscle-derived IL-6 will be elucidated.

## Data Availability

Not aplicable.
